# A case report on para-kala-azar dermal leishmaniasis: an unresolved mystery

**DOI:** 10.1186/s12879-023-08918-1

**Published:** 2023-12-18

**Authors:** Md. Mehedi Hasan, Sanghita Banik Proma, Md. Saddam Hossain, Md. Arifuzzaman, Naylla Islam, Md. Abu Bakar Siddique, Amiruzzaman

**Affiliations:** 1 Department of Medicine, Sir Salimullah Medical College Mitford Hospital, Dhaka, Bangladesh; 2 Government Employee Hospital, Dhaka, Bangladesh; 3 Colonel Malek Medical College, Manikganj, Bangladesh

**Keywords:** Para-kala-azar, PKDL, Post-kala-azar dermal Leishmaniasis, VL, Visceral Leishmaniasis

## Abstract

**Background:**

Post-kala-azar dermal leishmaniasis (PKDL) is a dermatosis that occurs 2–3 years after an apparently successful treatment of visceral leishmaniasis (VL). In rare cases, PKDL occurs concurrently with VL and is characterized by fever, splenomegaly, hepatomegaly or lymphadenopathy, and poor nutritional status and is known as Para-kala-azar dermal leishmaniasis (Para-KDL). Co-association of active VL in PKDL patients is documented in Africa, but very few case reports are found in South Asia.

**Case summary:**

We present a case of Para-kala-azar Dermal Leishmaniasis (Para-KDL) in a 50-year-old male patient with a history of one primary Visceral Leishmaniasis (VL) and 2 times relapse of Visceral Leishmaniasis (VL). The patient presented with fever, skin lesions, and hepatosplenomegaly. Laboratory tests revealed LD bodies in the slit skin smear and splenic biopsy. The patient was treated with two cycles of Amphotericin B with Miltefosine in between cycles for 12 weeks to obtain full recovery.

**Conclusion:**

This case report serves as a reminder that Para-kala-azar dermal leishmaniasis can develop as a consequence of prior visceral leishmaniasis episodes, even after apparently effective therapy. Since para-kala-azar is a source of infectious spread, endemics cannot be avoided unless it is effectively recognized and treated.

## Introduction

Leishmaniasis is one of the top ten neglected tropical diseases, with an incidence rate of 12 million, affecting 0.9 to 1.6 million new cases each year [1]. In tropical and subtropical regions like Bangladesh, the sand fly’s (especially sp. Leishmania Donovani) habitat, leishmaniasis, is considered a serious public health concern. Leishmaniasis manifests clinically in three distinct diseases. Visceral leishmaniasis (VL), where fatal inflammation of the liver and spleen occurs; destructive Mucocutaneous leishmaniasis (ML), where mucosal involvement coincides with cutaneous involvement or occurs after the clearance of cutaneous lesion [2], and Cutaneous leishmaniasis (CL) of skin which presents with ulcerative granulomatous lesion which heals spontaneously. Post-kala-azar dermal leishmaniasis (PKDL) is another intriguing form of leishmaniasis that often occurs as a sequela following an apparently effective treatment of visceral leishmaniasis [3], which presents with hypomelanotic, macular, papular, nodular, or mixed rash.

Usually, PKDL occurs as a separate event 2–3 years after the apparent cure of VL due to the reactivation of the dormant LD bodies on the dermis. The immunological mechanism of VL and PKDL does not support their co-existence. However, in a rare presentation, active VL can co-exist with PKDL. This co-association of active VL and PKDL is known as Para-kala-azar dermal leishmaniasis. Para-kala-azar dermal leishmaniasis (Para KDL) presents with a constellation of symptoms like fever, splenomegaly, hepatomegaly, or lymphadenopathy and poor nutritional status along with distinguishing features of PKDL rashes [4]. There have been a few documented cases of Para kala-azar dermal leishmaniasis (Para-KDL) from India [5], Iran [6], Kenya [7], and Bangladesh [8]. Due to the rarity of this disease, specific data regarding prevalence has yet to be established, although, the co-existence of active VL in PKDL patients has been documented in Sudan, with data from 16% of the overall sample size [9].

PKDL occurring concurrently with VL poses a diagnostic challenge for clinicians. The condition often gets misdiagnosed and treated arbitrarily without any successful outcome. Moreover, increasing resistance in multiple essential drugs like sodium stibogluconate [10], miltefosine [11] is a significant concern for the eradication of the disease. Given the crucial role of para-KDL in parasite transmission, the lack of information on its nature, etiopathology, and evidence based management protocol poses a significant threat to its potential rampant.

## Patient presentation

A 50-year-old non-diabetic male patient from Manikganj presented to Sir Salimullah Medical College Mitford Hospital in 2021 with fever, skin rash, and heaviness of the left upper abdomen for the previous three months. The fever was high grade (Highest recorded temp was 102 °F), intermittent in nature, was not associated with chills and rigor, and did not subside by sweating. The patients also complained of substantial weight loss despite having a good appetite.

On query, he gave a previous history of Visceral Leishmaniasis 20 years back and 2 histories of relapse of VL (Reappearance of symptoms and sign of Kalazar within 6 months after treatment) [12], one is 10 years, and another is 2 years back. He was treated with Sodium Stibogluconate the first two times and with Amphotericin B the last time. He had no other known illness.

**Fig. 1:**
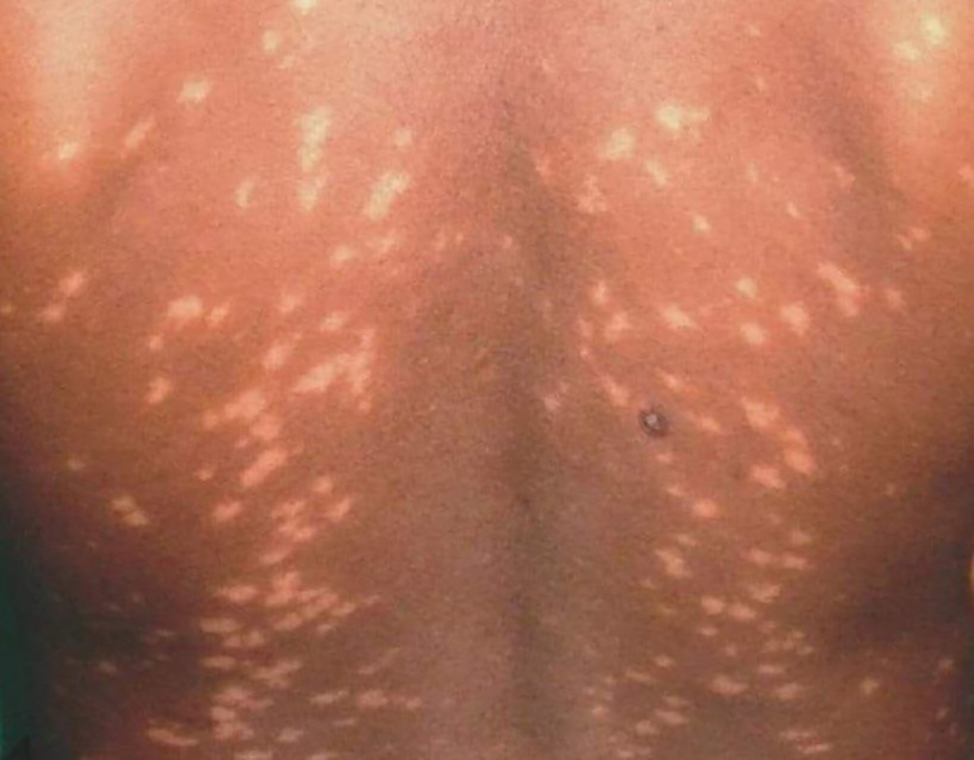
Multiple hypopigmented maculopapular rash present on the body, mostly on the back of patient

**Fig. 2:**
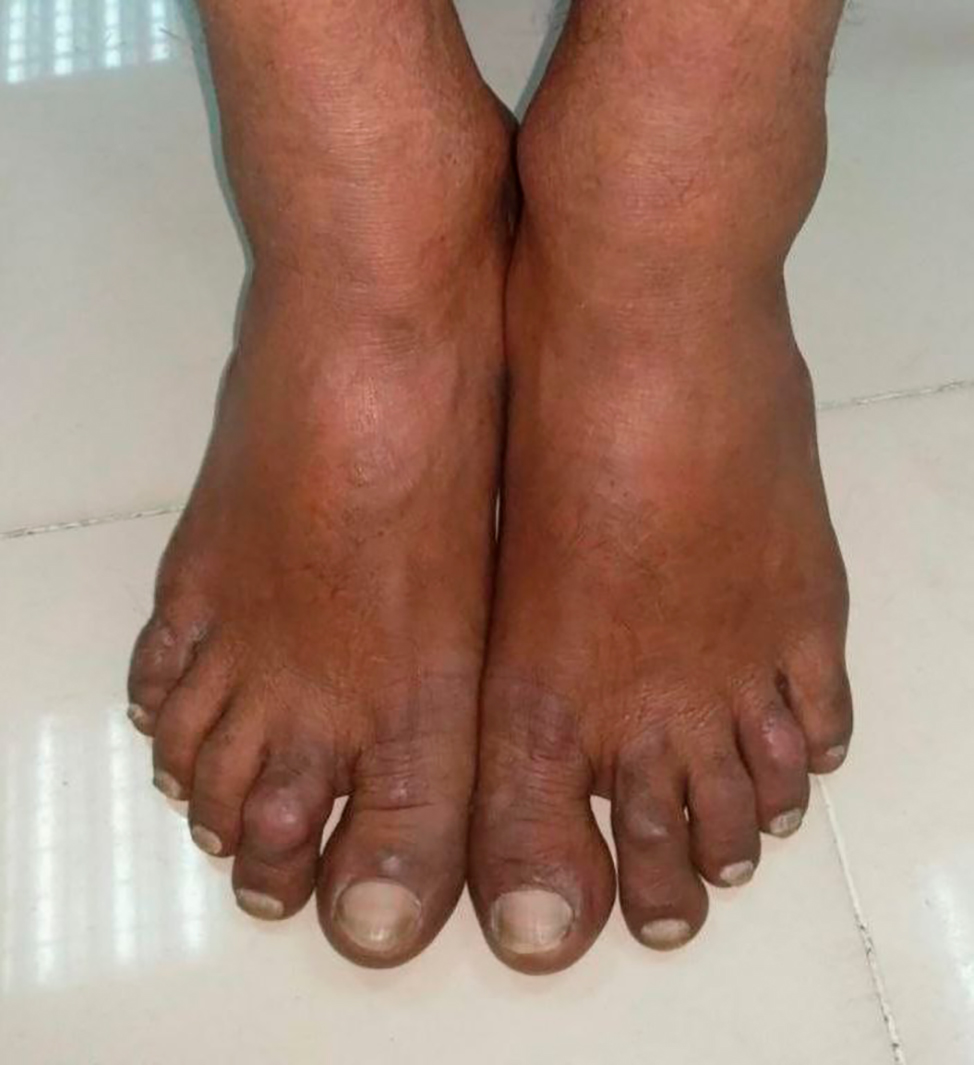
Erythematous rash on feet of patient

**Fig. 3:**
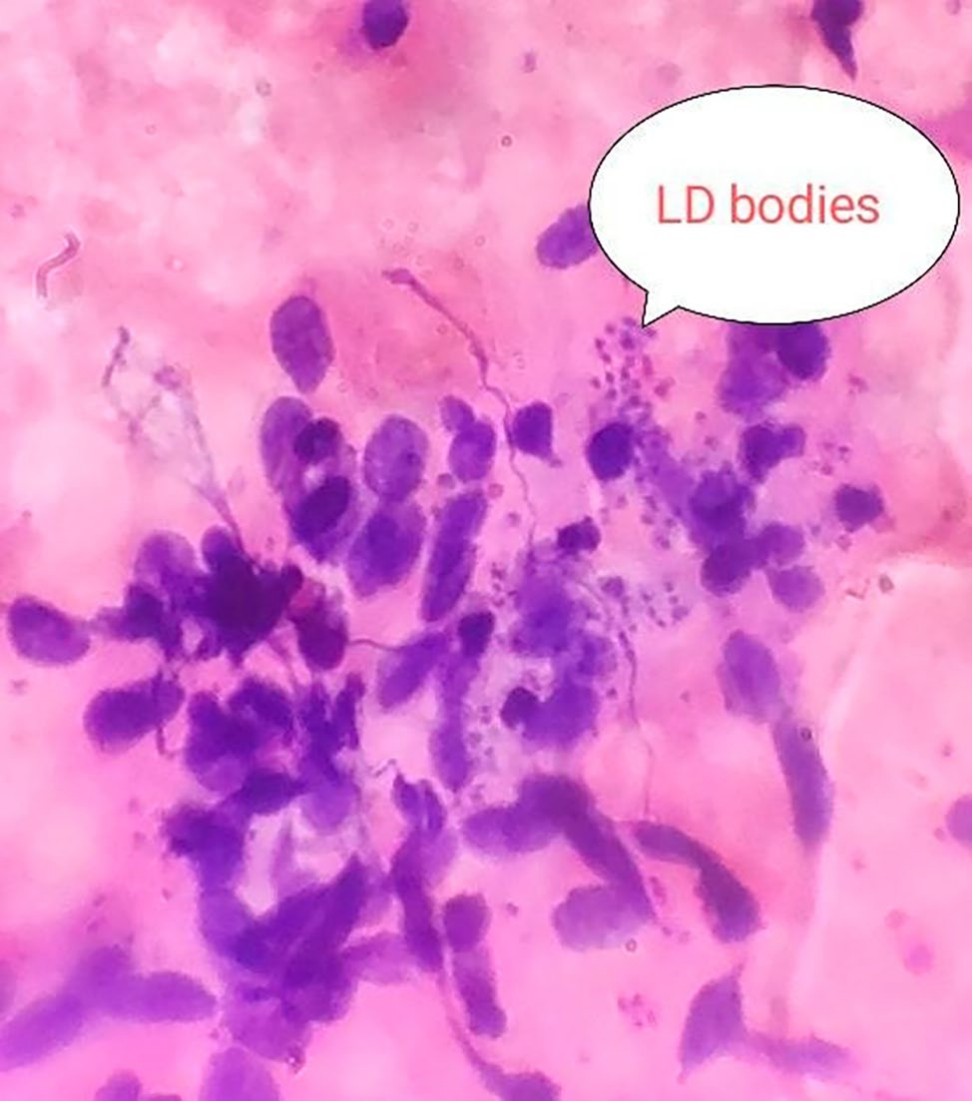
Splenic smear shows lymphocytes, histiocytes and blood. Leishmania Donovani (LD) bodies are seen within and outside the cytoplasm of histiocytes

**Fig. 4:**
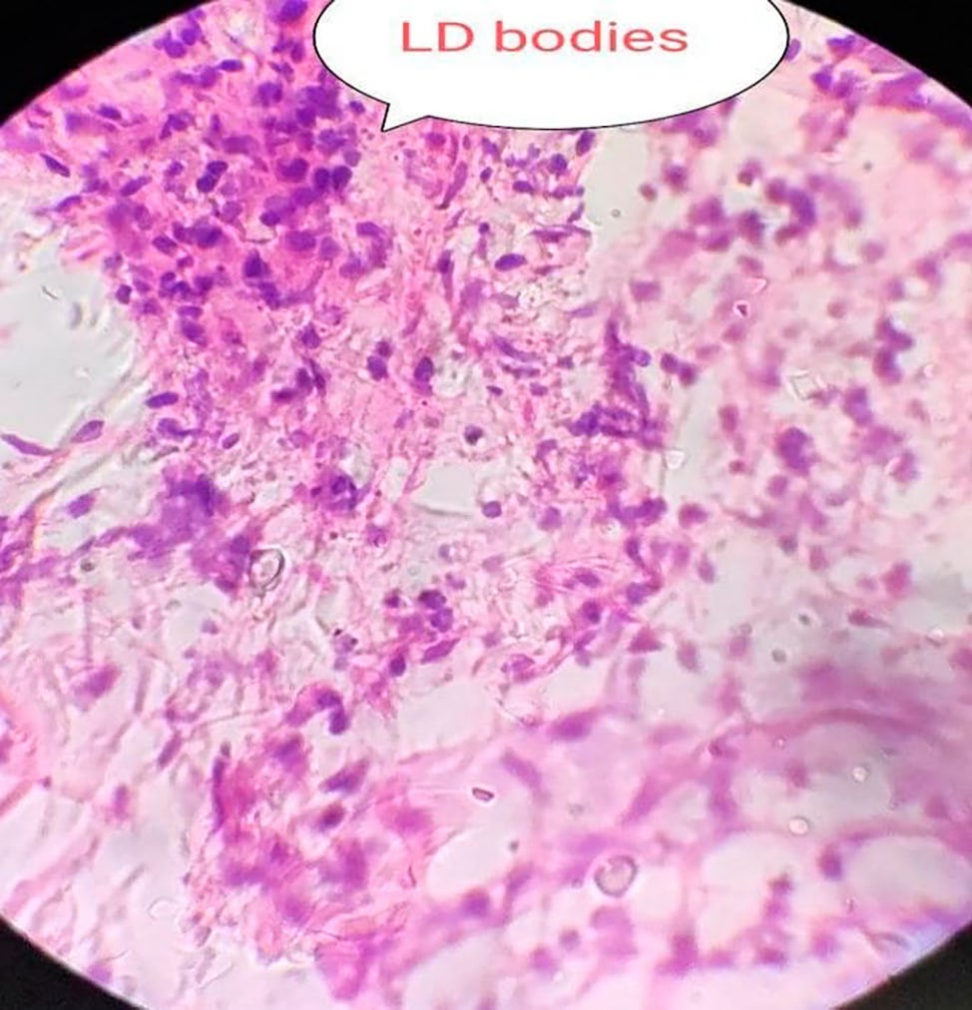
Slit skin smear section shows skin. The epidermis is thin and flat. The dermis reveals dense infiltration of histiocytes and lymphocytes, many of the histiocytes are packed with Leishmania Donovani (LD) bodies

### Clinical course

Examination revealed multiple maculopapular non-scaly skin lesions (Fig. 1) and a few erythematous lesions (Fig. 2) on the face, limbs, and back of the chest, ranging from 1 to 2 centimeters in diameter. Skin rashes developed gradually but remained non-itchy and non-tender,non-ulcerative, with no loss of sensation in those regions.

The left hypochondrium showed the most obvious signs of distention on abdominal examination. Significant splenomegaly (7 cm from the left coastal margin to the right iliac fossa) and hepatomegaly (2 cm from the right coastal margin) were present. Hemodynamically, the patient was stable. Hematological study revealed pancytopenia, including normocytic normochromic anemia (Table 1). Routine biochemistry revealed no abnormality (Table 2). But certain investigations showed specific findings conclusive of active kalazar (Table 3).

**Table 1:** Hematological study findings:

Parameters	Findings	Reference
Hemoglobin	9 g/dL	13–17 g/dL
ESR	130 mm/hr	0–12 mm/hr
White blood cells	1.53 K/uL	4–10 K/uL
Differential Count:		
Neutrophil	44%	40–80%
Lymphocyte	46%	20–40%
Platelet	61 K/uL	150–410 K/uL

*ESR = erythrocyte sedimentation rate

**Table 2:** Routine investigation findings:

Parameters	Findings	Reference
Urinary Routine Examination	Normal	
Serum creatinine	0.99 mg/dL	0.70–1.20 mm/dL
RBS	6.35 mmol/L	< 11.1 mmol/L
ALT	29 U/L	15–61 UL
ALP	120 U/L	38–126 U/L
Albumin	4.16 g/dL	3.50–5.20 g/dL
USG of whole abdomen	- Grade I prostate enlargement - No abdominal lymphadenopathy - No ascites	
Prothrombin time	16 s	12–17 s
Bleeding Time	3 min	2–7 min
Clotting Time	6 min	6–9 min
APTT	49.4 s	28–36 s
Uric Acid	6.25 mg/ dL	3.50–7.20 mg/dL
Calcium	10.15 mg/dL	8.80–10.60 mg/dL

*RBS = Random blood sugar, ALT = Alanine aminotransferase, ALP = Alkaline phosphatase, USG = Ultrasonogram, APTT = Activated partial thromboplastin clotting time

**Table 3:** Significant findings in different investigations:

Parameters	Findings
ICT for Kala-azar (Both from serum sample and splenic smear)	Positive
Bone Marrow Biopsy	Hyperactive erythropoiesis, active granulopoiesis and megakaryopoiesis. LD body was virtually absent.
Splenic Biopsy	LD bodies were present (Fig. 3)
Slit Skin Smear	Dense infiltration of histocytes and lymphocytes, many of the histocytes were packed with LD bodies (Fig. 4)

*ICT = Immunochromatographic dipstick test

All investigation reports and clinical examinations indicated the presence of active visceral leishmaniasis and PKDL simultaneously, leading to the confirmed diagnosis of Para kala-azar dermal leishmaniasis (Para-KDL).

Multiple documented cases reveal relapses linked to immunosuppression. However, our patient, despite experiencing relapse, did not exhibit any additional immunocompromised conditions or infections.

### Treatment module

**Fig. 5:**
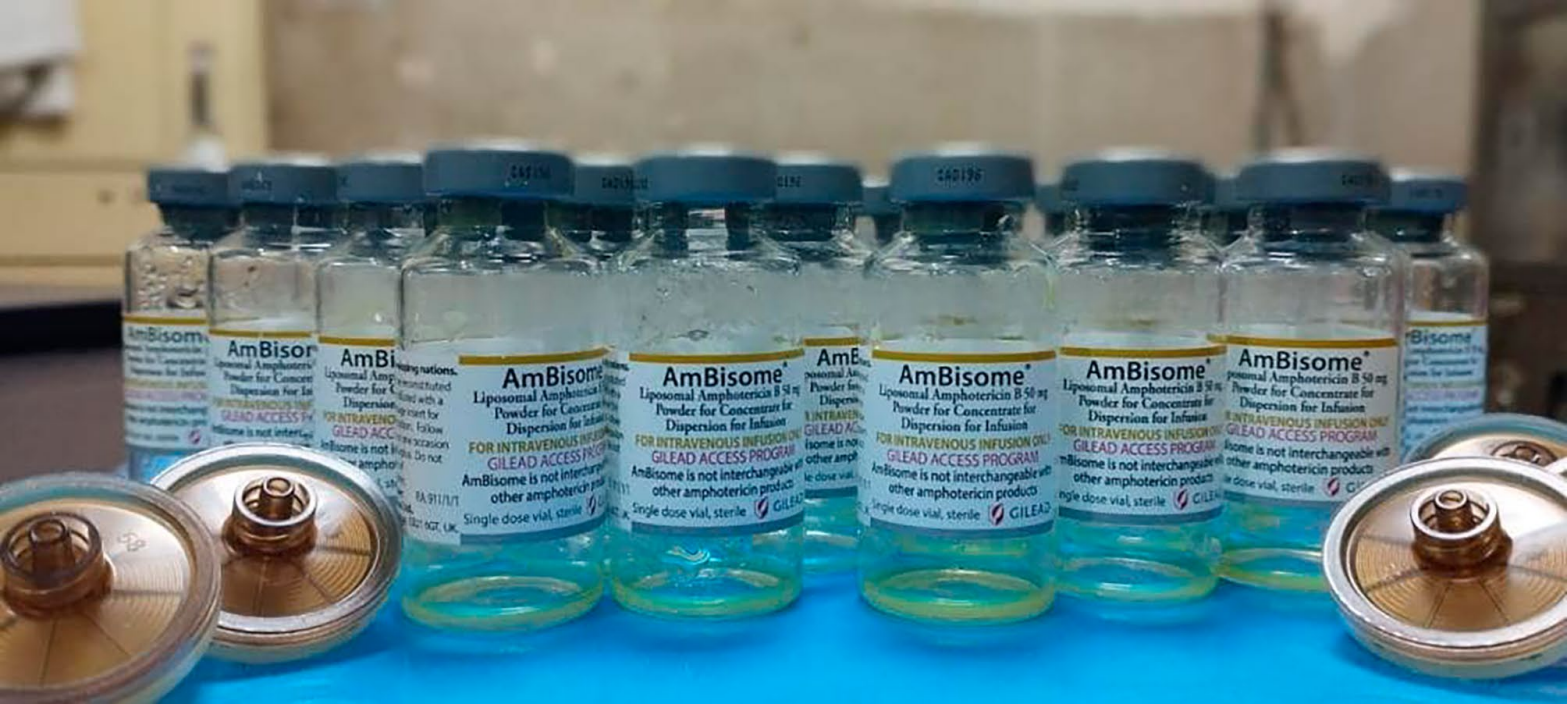
Empty vials of Liposomal Amphotericin B after administration

As per the National Guideline for Kala-azar Management in Bangladesh, the recommended treatment for visceral leishmaniasis (VL) involves a single-dose of Liposomal Amphotericin B at 10 mg/kg^12^. VL is more potent but treating post-kala-azar dermal leishmaniasis (PKDL) requires a higher dose and longer duration due to prolonged parasite survival [13]. In the case of our patient, who faced both the variants, a treatment protocol was planned from both the guideline and insights from previous reports. A case series was found from India where VL and PKDL were treated separately, utilizing liposomal amphotericin B for VL and initiating Miltefosine for PKDL one month after the initial VL treatment [14]. Our patient was also first treated with a single dosage of Liposomal Amphotericin B (10 mg/kg body weight) for relapse visceral leishmaniasis, followed by Miltefosine (2.5 mg/kg/daily) 50 mg twice a day for 12 weeks for PKDL (Fig. 5).

During a 3-month follow-up, the PKDL had improved, as seen by the disappearance of the skin rashes and the absence of LD bodies in the skin smear from the Slit incision. However, several systemic features, such as splenomegaly and failure to gain weight, were still evident. Thus, the patient was treated again with three doses of Liposomal Amphotericin B (5 mg /kg) on alternate days.

One month following the last dose of liposomal amphotericin B, we followed up with the patient again. This time, the patient’s overall clinical condition was improved. The patient had gained weight. There was no fever or splenomegaly. We followed up with the patient again at 6 months, and there was no fever, no splenomegaly, and the patient was feeling of general wellbeing. The patient was definitively cured [12]. The patient was also in good health during his follow-up after 2 years in 2023.

## Conclusion

Because para-kala-azar is a relatively uncommon diagnosis, we used our case to demonstrate why we should consider it a differential diagnosis when a patient appears with similar symptoms. As para-kala-azar continues to be an active source of its contagious spread, patients need to be appropriately evaluated and treated to avoid transmission. Recently, Bangladesh has been declared the first country to eliminate kala-azar, but it cannot be sustained unless all sources of infection are eradicated. With increasing parasite resistance in multiple drugs, we hope our case serves as a reminder of the significance of additional research required for the fight against kala-azar and improvement of case management strategies.

## Data Availability

All data are available on reasonable request to authors Dr. Sanghita Banik Proma (psanghita81@gmail.com) and Dr. Md. Mehedi Hasan (mehedissmcms@gmail.com).
